# Active and Intelligent Packaging: A Review of the Possible Application of Cyclodextrins in Food Storage and Safety Indicators

**DOI:** 10.3390/polym15214317

**Published:** 2023-11-03

**Authors:** Andrés Leobardo Puebla-Duarte, Irela Santos-Sauceda, Francisco Rodríguez-Félix, Rey David Iturralde-García, Daniel Fernández-Quiroz, Ingrid Daniela Pérez-Cabral, Carmen Lizette Del-Toro-Sánchez

**Affiliations:** 1 Departamento de Investigación y Posgrado en Alimentos, Universidad de Sonora, Blvd. Luis Encinas y Rosales S/N, Col. Centro, Hermosillo 83000, Mexico; a215201577@unison.mx (A.L.P.-D.); francisco.rodriguezfelix@unison.mx (F.R.-F.); rey.iturralde@unison.mx (R.D.I.-G.); a222230199@unison.mx (I.D.P.-C.); 2 Departamento de Investigación en Polímeros y Materiales, Universidad de Sonora, Blvd. Luis Encinas y Rosales S/N, Col. Centro, Hermosillo 83000, Mexico; irela.santos@unison.mx; 3 Departamento de Ingeniería Química y Metalurgia, Universidad de Sonora, Blvd. Luis Encinas y Rosales S/N, Col. Centro, Hermosillo 83000, Mexico; daniel.fernandez@unison.mx

**Keywords:** cyclodextrin, smart packaging, intelligent packaging, active packaging, food additives, biopolymers, polysaccharide

## Abstract

Natural cyclodextrins (CDs) can be formed by 6, 7, or 8 glucose molecules (α-, β-, and γ-, respectively) linked in a ring, creating a cone shape. Its interior has an affinity for hydrophobic molecules, while the exterior is hydrophilic and can interact with water molecules. This feature has been used to develop active packaging applied to food, interacting with the product or its environment to improve one or more aspects of its quality or safety. It also provides monitoring information when food is optimal for consumption, as intelligent packaging is essential for the consumer and the merchant. Therefore, this review will focus on discerning which packaging is most appropriate for each situation, solubility and toxicological considerations, characterization techniques, effect on the guest properties, and other aspects related to forming the inclusion complex with bioactive molecules applied to packaging.

## 1. Introduction

For some years now, and even faster with the passing of the pandemic (COVID-19), consumers have chosen to have food stored in their homes for easy access and, in case of an emergency, as a future food supply. Therefore, the time frame in which these packaged foods are no longer suitable for consumption is a concern [1,2,3]. Food packaging was developed to serve several purposes, such as limiting food loss and preserving food quality for extended periods. Its main functions can be summarized as protection against possible contamination (acting as a barrier), containment, communication of package information about brands and nutritional content, and convenience to adapt to the fast-paced customer lifestyle [4].

It is necessary to be aware of the conditions in which the food is stored, considering the appropriate temperature range and the suggested consumption date, to be fully informed of their quality concerning time [5]. This is where smart packaging (SP) comes in, which is a technology applied in the packaging system to extend shelf life and reduce food waste [6]. SP can be divided into active packaging (AP) and intelligent packaging (IP). AP interacts directly with the food, focusing on incorporating food additives to prevent or delay deterioration. It considers food factors such as respiration rate, humidity, and microbial attack to maintain a remarkable quality by reacting against unfavorable components. On the contrary, IP does not directly interact with the food; it only provides real-time information on the state of the food, indicating whether it is still optimal for consumption, always working in the distribution chain, from the industrial plant to the consumer’s home [5].

Therefore, researchers and industries have focused on finding efficient ways to achieve this, designing them based on polymers, improving the characteristics of foods without affecting their sensory properties, synthesizing molecules with complex structures capable of stopping catalytic reactions or even slowing them down, and assembling with the ‘problem molecules’ by trapping them inside their cavity. This can be achieved, for example, with inclusion complexes, with a ‘host’ and a ‘guest’ molecule [7,8,9].

Inclusion complexes are aggregates of molecules stabilized via non-covalent bonds (for example, van der Waals, hydrogen bonds, and hydrophobic interactions) [10]. Host molecules are characterized by having an inner cavity where another molecule, usually referred to as a “guest molecule”, can be incorporated [11]. Therefore, hosts will act as receptors and guests as substrates, inhibitors, or cofactors [12]. The resulting molecular inclusion complex can easily break down in determined physiological environments [13]. These inclusion systems can improve the physicochemical properties of the molecules hosted in the cavity, such as solubility, dissolution, absorption, bioavailability, and biological activity [14,15]. Various inclusion complexes have been synthesized, including cyclodextrins, both modified and native, highlighting the latter as the most used [16].

Our article includes the most recent studies on food packaging using the CD, which have presented essential applications. Over the last decades, critical reviews such as Pereira et al. have developed [17], reviewing the latest advances in the application of CDs and their current legislation. The main difference between their study and ours is that they focus on the general sensory qualities of all varieties, both native and modified, without indicating their direct (active packaging) or indirect (smart packaging) interaction with food and not highlighting the disadvantages of their application. More recent studies, such as Zhou et al. [18], do not highlight the organoleptic qualities enhanced by this packaging method. This study sheds light on the improved properties of CD and the differences in the applications of the two packaging methods (active and intelligent), discerning their application disadvantages.

This review provides an overview of the published works about active and intelligent packaging involving CDs, along with a discussion of the diverse applications given in the industry, highlighting the focus of each analyzed study and the enhanced properties of the guest molecule. The review was based on articles published between 2018 and 2023.

## 2. Cyclodextrins

Cyclodextrins (CDs) have piqued the scientific community’s interest due to their structural characteristics, for which various applications have been found. CDs are cyclic oligosaccharide structures composed of α-1,4 D-glucopyranoside bonds with a hydrophobic core inside and a hydrophilic outer surface due to the position of their hydroxyl groups [19,20,21]. They can be differentiated by the number of glucoses that present their α, β, and γ structures. The main differences are found in Table 1. The main characteristic that distinguishes CDs is their ability to easily interact with water due to their hydrophilic surface, which presents good solubility, and their interior hydrophobic cavity, which forms inclusion complexes with lipophilic molecules [22,23,24,25].

To know which CD is most likely to encapsulate a guest inside its cavity, the size of the hole must first be evaluated to see if host-guest interaction is possible. A-CD has the most minor cavity. Therefore, it cannot accept some large molecules. Γ-CD presents the largest cavity, being able to encapsulate larger molecules. A-CD and γ-CD present the highest solubilities in contrast to β-CD, which has an intermediate-size cavity and poor solubility compared to the first two (Table 1). However, the most widely used due to its encapsulation performance is β-CD, added to the fact that it can encapsulate molecules such as lipids, vitamins, and other hydrophobic compounds [26,27]. Furthermore, it has the most outstanding strength due to hydrogen bond interactions, which provide greater interaction strength with the host molecule, avoiding its early release upon application [28]. Β-CD consists of 7 glucopyranose units in the shape of a truncated cone [29,30,31,32]. It has been widely used as an encapsulant or carrier of food additives because it improves the water solubility of the guest component, as well as its permeability, and provides stability to lipophilic compounds thanks to the hydroxyl groups that allow it to form hydrogen bonds (weak bonds), resulting in its nonpolar character [33,34,35,36,37].

**Table 1:** Main properties of the three native cyclodextrins. Created based on information from [38,39,40].

Physicochemical Properties	α-CD	β-CD	γ-CD
Chemical formula	$C_{36}H_{60}O_{30}$	$C_{42}H_{70}O_{35}$	$C_{48}H_{80}O_{40}$
Glucose units	6	7	8
Molecular weight (Da)	972	1135	1297
Internal diameter (nm)	0.47–0.53	0.60–0.65	0.75–0.83
Outer diameter (nm)	1.46	1.54	1.75
Height of torus (nm)	0.79	0.79	0.79
Internal volume (${nm}^{3}$)	0.174	0.262	0.427
Solubility in water at 25 °C (mg/mL)	145	18.5	232
Internal water molecules	6–8	11–12	13–17

## 3. Solubility and Toxicological Considerations

CDs have high solubility in water because their hydrophilic outer part is polar, possessing the ability to form stable emulsions due to the difference in polarity with their cavity [22]. Solubility is a quality that can be attributed to CDs, making it a significant application option within packaging systems since it can release hydrophobic host molecules found in its cavity in the aqueous phase.

CDs have numerous uses in different industrial areas, such as chemistry, pharmaceuticals, food, etc. In the pharmaceutical field, native and modified CDs have been utilized as a drug release system due to their high solubility, easy dilution, and ability to improve the physicochemical properties of the guest molecule while maintaining the conditions specified on the package, such as effectiveness and purity, for established periods [41,42,43,44,45]. Considerable improvements in active pharmaceutical ingredients (APIs) have been observed, presenting greater solubility in water, effectiveness, and physical and chemical stability [46]. CDs have been used for some years in the chemical industry to increase the solubility of hydrophobic molecules; such examples are essential oils in perfumes [47]. Specifically for food, CDs have been employed to take care of the organoleptic properties, increasing the shelf life of products and wholly or partially eliminating odors, flavors, and unwanted compounds. They are also applied as aroma stabilizers and increase the solubility of vitamins and lipids in aqueous systems [22,48,49,50,51,52,53,54,55].

These processes of entrapment of molecules with a hydrophobic character are reversible when the inclusion complex encounters a solvent. In response to this interaction, the molecules are released into the medium in which this solution is found [17].

Consequently, the CDs have obtained some recognition, namely their entry to the GRAS list (a list of food additives by the Food and Drug Administration that recognizes them as safe) and their approval by the European Medicines Agency (EMA), which allows their commercialization with a certain degree of purity [56]. The World Health Organization (WHO) recommends a maximum of 5 mg/kg per day as a food additive. It is essential to consider this when devising an active container that will be in contact with the food and could be ingested by the consumer. The Environmental Protection Agency (EPA) also ruled out the need to have maximum permissible levels for the residues of the three main native CDs (α, β, and γ). Therefore, it is concluded that, according to all the available studies, CD presents an almost insignificant toxicity [57,58,59].

## 4. Formation of Inclusion Complexes

The formation of inclusion complexes has been studied using analytical techniques to determine the possible structures that encapsulation can form [60]. Inclusion complexes are formed when the host molecule, with the correct size for absorption, is positioned within the CD cavity. It should be mentioned that CD has the possibility of including both hydrophobic and hydrophilic molecules in its structure [40,54,61]. Once encapsulated, the hydrophobic molecule can increase its water solubility and bioavailability [62].

There are some parameters to consider before carrying out the encapsulation or packaging of the molecule inside the cavity of the CDs. The size of the cavity is essential to knowing if the host molecule could habituate in it. Table 1 shows the respective measures for each native CD. In addition, it should also be considered that CDs crystallize by two mechanisms, which depend on the guest molecule and the CDs that form the complex. This will result in one of the categories of the crystal packing phenomenon resulting in channel or cage structures [40].

**Figure 1:**
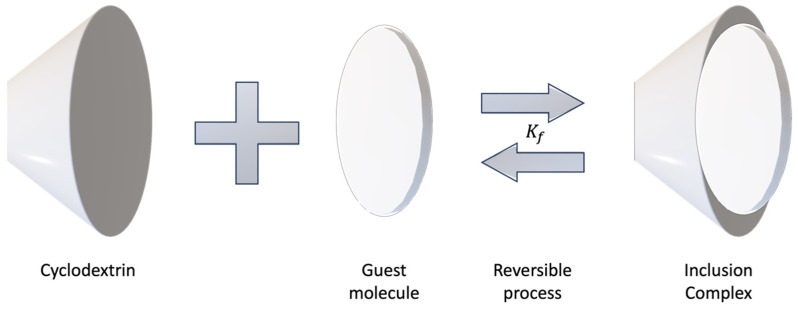
Schematic illustration of the formation of an inclusion complex.

Inclusion complex formation is governed by the equilibrium association/dissociation constant for the host molecule, CDs, and inclusion complex [63]. Because weak interactions govern CDs, the encapsulated host can break free of its environment without much effort [40]. The higher the formation constant ($K_{f}$) for this reversible process (Figure 1), the more stable the inclusion complex will be and the less in favor of dissociation [35,38].

In the same way, the formation of inclusion complexes is also strongly affected by interaction forces such as hydrogen bonds, van der Waals forces, and electrostatic and hydrophobic interactions [10,35,36]. In other words, to increase the entropy of the system, the water molecules located in the CD cavity are displaced by the hydrophobic molecules to form an apolar–apolar association, achieving better energetic stability for the complex [34].

### 4.1. Inclusion Complex Formation Techniques

To determine the appropriate encapsulation method, it is necessary to consider the physicochemical properties of the host and guest molecules to increase the performance of the inclusion complex, as well as its release rate and bio-accessibility [64]. Mentioning some of the industrial techniques for their large-scale productions and low temperatures for their preparation, we can highlight spray drying, supercritical fluid, and freeze-drying. However, they present a high production cost and require more sophisticated equipment [38,65]. We also highlight two other techniques, the precipitation method and physical mixture (known as kneading), as being the most accessible at the laboratory scale due to their simplicity and high performance [56,66], which we will detail below.

#### 4.1.1. Precipitation Method

It is the most widely used method due to its ease and efficiency at the laboratory level. It is also used when the host molecule is insoluble in water. First, the CD is placed in an aqueous solution where it is heated until it dissolves in the medium, and if the guest molecule withstands the dilution temperature of the CD, it is added at this moment. Subsequently, it is shaken to provoke the interaction between host and guest, which can be in refrigeration or at low temperatures. 

Lastly, the solution is filtered and dried to include complex powder [18,40]. To mention some studies with this methodology, we can highlight the preparation of a container based on lutein and β-CD with possible applications as IP or AP [67].

#### 4.1.2. Physical Mixture

It results in a simple method with low encapsulation yields in which a mass can be obtained instead of a fine powder. This method consists of adding a small amount of water to the host molecule to form a paste (slurry), followed by adding the guest molecule. Afterward, kneading is applied until a uniform mixture is formed. The kneading time required will depend on the host molecule [40]. Some studies elaborated with this methodology: preparation of a physical mixture from naringenin and β-CD in a mortar and refrigerated at 4 °C [68] and the elaboration of nanocomposites from zein/catechin/β-cyclodextrin as AP [69]. 

### 4.2. Characterization Techniques

**Figure 2:**
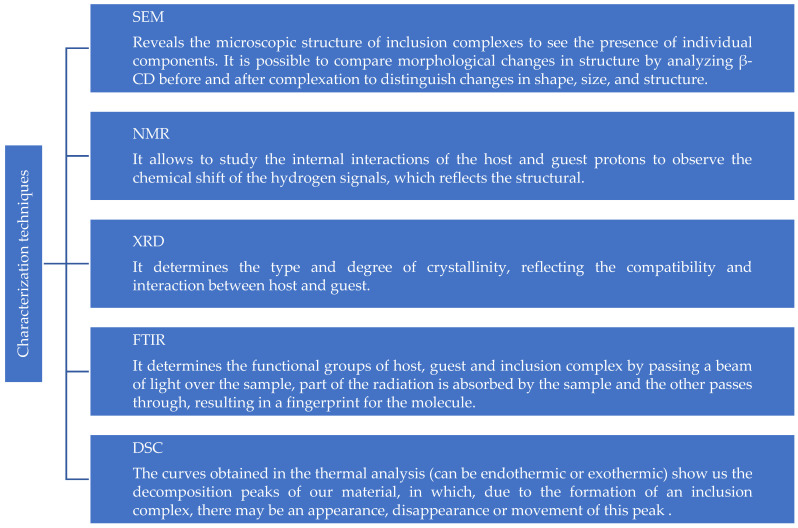
Characterization techniques to confirm the formation of a package [40,72,73,74].

We can characterize the sample obtained to confirm that the structure of an inclusion complex was formed. The most common chemical-structural characterizations are thermal analysis, Fourier transform infrared spectroscopy (FTIR), nuclear magnetic resonance (NMR), and X-ray diffraction (XRD) (Figure 2) [62,70,71]. Observing any conformational change in the different chemical-structural studies, we can assume that a new polymer has been formed, or, better said, an inclusion complex (Table 2). Thanks to the complex formation properties of β-CD, we can generate different applications in food to protect hydrophobic molecules (non-polar characters) such as oils, vitamins, lipids, and fats, serving as smart or active packaging for these molecules.

### 4.3. Effect on the Guest Properties

As we have seen, CDs improve the stability of their guest molecules, promoting their resistance to temperature, light, and oxygen, among others. It protects against different factors, masks unwanted odors and flavors, and contributes to a prolonged release, making it a viable option for an SP since it is presented as an alternative to solve food quality problems. In this way, food packaging plays a vital role in mitigating food waste and caring in a certain way for the planet on which we live.

#### 4.3.1. Protection

CDs generally protect molecules in their cavity, including essential oils, lipids, vitamins, flavors, and colors [22]. In the same way, its protective function is not only based on caring for the attributes of food but also protecting from molecules that are naturally included in food, an example of which is the removal of cholesterol from dairy products [86,87], where it was observed that it effectively removed cholesterol, obtaining efficiencies of over 95% for milk, cream, butter, and cottage cheese. Additionally, a decrease in aflatoxin, a toxin present in these dairy products associated with the extraction of cholesterol from the packaging, was found without affecting its textural properties. 

Likewise, volatile phenols from wine [88], where an instantaneous removal of 45 to 77% of the phenolic compounds present as unwanted odors in heavily tainted wines was obtained, and mycotoxins such as alternariol (AOH) from cereals, tomatoes, grapes, and other susceptible fruits were removed by packaging this mycotoxin, which, with prolonged exposure, can cause cancer. β-CD, γ-CD, and CD polymers were used for packaging this potentially damaging molecule. More stable complexes were formed, with γ-CD presenting higher fluorescence [89]. It has also been found that β-CD-based AP effectively protects dry food products, demonstrating antimicrobial properties and delaying mold growth in wheat grains, maintaining their average germination properties [90].

#### 4.3.2. Taste Modifications

When food is stored for long periods, its odors and flavors sometimes begin to decrease or become unpleasant. This is where CDs come in with their ability to encapsulate and stabilize their smells and tastes, preserve food for extended storage times, and protect it from heat treatments, such as freezing or thawing in microwaves, which can somewhat degrade food compounds [40,91]. Some studies have used CD packaging to enhance flavors or delay degradation. Such is the case of the use of β-CD, which resulted in the thermal stabilization of the formation of Amadori products and decreased the degradation constant, slowing down the appearance of brown-colored compounds and enhancing good flavors [92]. Likewise, determinations have been made to standardize the degrees of spiciness in sauces and other products, such as the case where an electrochemical sensor was built based on a multiwall of carbon nanotubes, incorporating β-CD/carboxylated, and had effective results thanks to the fact that the CD caused the dispersion of the nanotubes, achieving the detection of trace contents of capsaicinoids in soy sauce and meat products [93].

Another example of a study with good results is on the influence of yeasts and CD for preservation through the packaging of the bioactive compounds that give flavor to a red apple-based cider drink, where it was obtained that the β-CD significantly influenced the packaging of these compounds, achieving flavor retention of up to 18% during storage time [94]. It should be noted that AP cannot be designed to reduce or eliminate the presence of unwanted molecules, especially if these molecules are products formed at the end of their storage. This could result in consumers consuming undesirable flavors, odors, or deteriorated products. Therefore, IP could be an alternative to keep consumers informed of the product’s status, indicating when it may be suitable for consumption. That is why it is so important to study it.

## 5. Cyclodextrins in Food Packaging

There has been an unceasing interest for a long time in preventing food spoilage by increasing its shelf life and controlling spoilage reactions such as the action of polyphenol oxidase (PPO) and enzymatic browning reactions. This is why microbial inhibition mechanisms, among others, have been studied to increase the shelf life of food [70,95,96,97,98]. Many of the food packaging commonly sold in stores contains compound polymers derived from the petrochemical industry (low-density polyethylene, polyethylene terephthalate, and polypropylene, among others) that have the possibility of migrating into the food, altering its quality. These polymers also impact the environment due to their difficulty deleting [99]. Therefore, the development of more environmentally friendly packaging has become a necessity.

Consumers are looking for more natural and organic foods that meet specific characteristics, including their nutritional needs and being regarded as healthy. That is why scientists and researchers have focused on developing new technologies that meet these requirements, including additives, treatments with modified atmospheres, and intelligent and active packaging techniques to preserve their nutritional value. Encapsulation is presented as an advantage to meet the needs requested by consumers, stabilizing the compounds present in food that cause their degradation, oxidation, and unpleasant flavors and odors, as well as improving sensory quality. It is a technology for packaging solids, liquids, and gases to be released under specific conditions, considering that they can also be affected by external factors such as temperature, light, humidity, etc. [81].

Intelligent and active packaging are emerging technologies that are presented as a great potential option to erase the problem or somehow delay the deterioration of natural and everyday foods consumed, further satisfying the needs constantly changing within the international market. The benefit of taking advantage of these packaging systems lies in prolonging the shelf life of food products, proving them safe to consume, and simultaneously reducing food waste and negative environmental impacts [100]. Active packaging that comes into contact with food releases, emits, absorbs, or scavenges substances directly or indirectly into the food to maintain quality or delay degradation. Intelligent packaging acts as an indicator that provides information on the quality of the product without coming into direct contact with the food. It provides information to the consumer about the condition of packaged food using materials that monitor and interact with the package’s environment through an internal or external indicator.

It has been observed that when molecules are encapsulated in β-CD, they increase their stability when subjected to light, temperature, and oxygen. Still, they can also be used to hide the unpleasant flavors of some biomolecules [101,102]. Likewise, multicomponent encapsulations are sometimes carried out using a third molecule that can affect the stability and interaction between the CD and the guest molecule [103,104].

### 5.1. Active Packaging

Food packaging has a vital role within the supply chain as a precursor to maintaining food integrity in perfect conditions and meeting consumer demands for foods with higher quality standards [5,6]. An example of this is fruit. When fruits receive cut or minimal processing, they lose water, favoring microbial growth and oxidative reactions that generate bad-tasting compounds [105]. Therefore, active packaging technology emerges as an application strategy to improve food safety [106]. Studies show that packaging with antimicrobial properties comes into contact with food to reduce, slow down, or inhibit the growth of microorganisms that damage food [107]. 

With this, interest has been aroused in the development of active packaging that stops the microbial attack of the fruit, as is the case of packaging based on a film of polylactic acid (PLA) and β-cyclodextrin and allyl isothiocyanate inclusion complexes (AITC), where the results showed that the incorporation of a high concentration of this AP gave a more humid and polar surface in the PLA films, encouraging the diffusion of water through the matrix when it was immersed in a fat food simulant [108].

Like this essential oil, CDs can serve as a container for hydrophobic molecules such as lipids and vitamins [26]. Similar studies have been done with other CDs to supplement vitamins for people with diseases or disorders that do not allow the correct processing of hydrophobic compounds through potential active packaging used as drug delivery technology that increases their solubility. Such is the case of the deficiencies presented by people with fibrosis due to poor digestion, insufficient vitamins, and poor absorption. Therefore, they incorporated vitamins D3 and E into γ-CD, improving its bioavailability and stability [109].

Goñi-Ciaurriz et al. [110] improved the functionality of nanoparticles (NPs) with CDs, packaging sorbic acid (SA), and benzoic acid (BA) to be used as preservatives in food due to their antifungal and antibacterial properties, finding the association constants for this inclusion complex low. However, they obtained a high loading efficiency of these acids and had a prolonged release profile, thus achieving an inhibition system from the AP with CDs. Likewise, packaging has been manufactured with β-CD and carbon quantum dots composite nanoparticles (CQDs), improving the antioxidant activity and increasing the efficiency of naringenin encapsulation. Checking the formation of the active container through chemical-structural characterization by XRD, the formation of the complex was found by the change from crystalline to amorphous structure, indicating that an AP based on CD is indeed formed [68].

Other molecules with great antioxidant activity are carotenoids such as β-carotene, lycopene, and lutein, which present instability and can often undergo isomerization and decomposition due to oxidation. With the help of CDs, this damage can be slowed down, encasing it in its interior cavity. In another study, a possible application of an AP based on β-CD and lutein was performed, where the encapsulated lutein was guaranteed to protect its antioxidant properties [67]. Other plant pigments, like carotenoids, are flavonoids with antioxidant activity. However, their low solubility in the aqueous phase limits them in the food field. In one study, γ-CD was used as a package to protect it from photo-oxidation. Thanks to its application, the solubility increased 100 times compared to the pure extract of quercetin, significantly improving its antioxidant activity and resulting in a more significant inhibition of free radicals [111]. This opens the possibility of using the quercetin:γ-CD complex as active or smart packaging.

Mizera et al. developed composites made with linear low-density polyethylene (LLDPE) and β-CD:D-limonene, allowing them to avoid the loss by evaporation of the volatile compounds of this terpene by subjecting them to high temperatures and mechanical shearing processes, allowing them to be used for their antibacterial properties. CDs have also been used to perform extractions of bioactive compounds due to their ability to encapsulate in their cavities, acting as containers [80]. Vhangani et al. used β-CD to package raw green rooibo. It was shown that the increase in the concentration of β-CD improved the extraction yield of flavonoid polyphenols, increasing the antioxidant activity. Moreover, higher temperatures could be applied in the extraction when carrying out the packaging, avoiding degradation [112]. Likewise, essential oils have acquired a reputation for having good antioxidant activity; however, since they have high volatility and chemical instability, their applications in the food industry are limited.

Wu et al. made active packaging based on cinnamon essential oil (CEO) with five varieties of β-CD. In general, reasonable control over its release was observed, increasing its antioxidant and antibacterial activity and presenting itself as an alternative for storage [98]. Encapsulation in modified CDs affects the solubility, stability, and bioactive properties of the packaged compounds in different ways, but in all the cases studied, the solubility of essential oils increased [98,113,114]. This demonstrates CD’s protective role as an active container for essential oils through encapsulation, protecting their antioxidant and antimicrobial properties. However, improving its poor solubility in aqueous phases is important, which limits its application in the food industry.

Christaki et al. [75] encapsulated sage essential oil and β-CD, showing satisfactory values for encapsulation efficiency and correct inhibition for *S. aureus* and *L. monocytogenes* [75]. These results indicate that it may be possible to apply an AP to extend the shelf life of food to stop microbial attacks. Other guest molecules packaged in CDs with powerful chemical effects on resistant bacteria have been elaborated to verify their antibacterial properties and possible application in the food sector.

Li et al. packaged benzyl isothiocyanate (BITC) in β-CD, finding its significant bactericidal effect on *Escherichia coli* and *Staphylococcus aureus*. Subsequently, it was evaluated in broccoli juice, demonstrating stability and controlled release by BITC [115]. Finding ways to mitigate microbial contamination of food is crucial as it threatens the consumer. Until now, active packaging has been presented as an excellent option to control this problem [22]. Numerous investigations have been carried out on the applications of CDs, using them as active packaging, thus improving sensory properties, extending shelf-life, and Pickering emulsions (Table 3).

Liu et al. [125] made a Pickering emulsion as a carrier based on β-CD and cinnamaldehyde (CA) to be applied to different types of oil, protecting the lipids located in the emulsions against temperature and providing a protective shell. This potential AP was confirmed to have good storage stability, a pleasant taste, lower malondialdehyde (MDA) content, and antioxidant activity. Foods that contain more lipids can promote the faster formation of bad-tasting compounds and unwanted odors due to their ease of oxidation compared to other foods [126]. With this said, and knowing that CDs have this amphiphilicity ability, CD becomes a great option as a stabilizer for emulsions.

Another widespread use of active packaging is as a coating or film for meat foods, where they are strongly influenced by their high moisture, fat, and protein content, resulting in microbial solid attack and causing food spoilage [97,127]. Wu et al. designed an antibacterial film based on active packaging of curcumin and β-CD that could extend the shelf life of chilled pork, inhibiting microbial growth and lipid oxidation during storage. Likewise, there was a notable improvement in the coloring. This study lays the foundations for applying this active packaging to other meat systems, preventing mass loss due to decomposition [97].

Food preservation through an active packaging system is presented as an advantage to meet consumers’ needs and reduce food waste, thus completing the sustainable development goals (SDGs).

### 5.2. Intelligent Packaging

Plastic materials have strongly impacted the food packaging sector due to their low cost, durability, and different mechanical barrier properties (optical, rheological, and transport) [128,129]. In addition, to mention a few examples, they promise, through food packaging, protection against oxidation, humidity, light, and microbial contamination [130,131].

Food safety consists of keeping food away from unsafe conditions that endanger its security. Food quality and safety significantly impact consumers, acting as indicators that guarantee the freshness of food. Since freshness is manifested as a need by consumers, it has become a priority. Because of this, different technologies have been developed to let the customer know that their food continues to preserve its original quality [132,133].

Intelligent packaging is an improvement on the traditional packaging of the food industry with the implementation of sensors or indicators to inform the consumer of the state of the food, detecting changes in its initial conditions and indicating its state in real-time [130,134,135]. In this way, consumers obtain a better shopping experience, avoiding spending on foods that do not meet their ideal characteristics and preventing food waste (Table 4). Consumers generally use the shelf-life information (expiry date) to determine the level of freshness and quality according to the proximity to this date embodied on the package [136]. However, some foods with a “best-before date,” such as fruits, vegetables, and meat, are not as reliable due to the changes they experience since their production or harvest.

The primary and only function of intelligent packaging, which acts as sensors or indicators, is to measure any alteration of the initial conditions or conditions in which the food was offered to the consumer, responding to different stimuli, visualizing through the change in the intensity of a color scale, and determining the presence or absence of foreign matter inside the container [148,149,150,151]. The indicators are added to the food packaging as a visible label that, according to the variations presented by the food, will indicate the quality status for consumption [5]. In intelligent packaging, we can highlight the application of CDs in some indicators or sensors: leak indicators, freshness indicators, pH indicators, and electrochemical sensors (Table 2).

#### 5.2.1. Gas Indicators

These indicators, or sensors, show the quality of the food according to the atmosphere contained within the container. The alterations can be caused by enzymatic reactions of the food or by the diffusion of gases through the container wall, and there may be variations in the optimal concentrations for storage [152]. Within the food distribution chain, there is the possibility that the packaging may be damaged and cracks may be generated that compromise its integrity and, therefore, affect the quality of the food. An example is foods susceptible to oxidation, such as oils, vitamins, and lipids, impacting microbial growth and the appearance of unwanted odors and flavors. The reagents that give colorimetric scales as oxygen input indicators are the most commonly used, governed by oxidation–reduction reactions and having simple manufacturing processes.

Nevertheless, these reagents present high instability because of their easy degradation in the presence of oxygen. Some of these indicators apply photocatalytic nanoparticles (NPs) to achieve the oxidation–reduction stability of some dyes, such as methylene blue. Jarupatnadech et al. designed intelligent packaging based on chitosan and montmorillonite packed in β-CD with methylene blue/glucose. Methylene blue was reduced to its colorless form by glucose and turned blue on exposure to oxygen. Films based on these polymers demonstrated storage stability at low temperatures, which makes them an excellent option for cold-stored food products due to their effectiveness as colorimetric oxygen indicators [137].

#### 5.2.2. Freshness Indicators

There are two different types of freshness indicators: direct and indirect. As the name implies, direct freshness indicators detect analytes in the food to indicate its condition. The indirect ones are based on reactions triggered by the degradation of food due to factors such as time, temperature, or pH [5,153]. Zhang et al. developed an intelligent film using PVA, β-CD, and acylated anthocyanins. The PCRA film presented good mechanical properties, stability, and sensitivity to color change, slightly lower than normal anthocyanins. However, the color of the PCRA film changed from pink to yellow/green, indicating that it can satisfactorily indicate beef freshness. In addition, they found a high correlation between the physical chemistry of meat and the information from the colorimetric film, demonstrating its potential application as an intelligent sensor for meat foods [139]. PVA has also been used in a film, as in the case of Lin et al., where they developed an SP based on PVA, chitosan in a container of curcumin:β-CD, as a freshness indicator for observing and maintaining pork and shrimp. The intelligent packaging of curcumin:β-CD improved the antioxidant and antibacterial activity, water vapor permeability, and mechanical properties of the PVA/chitosan film. The results are promising for its potential application as intelligent packaging [140].

#### 5.2.3. pH Indicators

Compared to other smart packaging, colorimetric pH indicators accurately provide deterioration and safety information [154,155]. These innovative packages present changes in their color scale when there is any alteration in the food [150]. An example of this is products with a high protein content, such as meat, where microbial growth or oxidation can be triggered by contact with the environment, activating the intelligent pH packaging. Due to its activation and these characteristics, it is considered one of the best options as a smart indicator [156]. Eze et al. developed a colorimetric film based on chitosan and broken riceberries. The results showed increased hydrophobicity, thermal stability, and antioxidant activity. In addition, an easily observable colorimetric response was obtained when it was applied to fresh shrimp, changing color from red/orange to yellow to respond to its deterioration, presenting itself as a feasible option for foods with similar conditions. When colorimetric pH indicators are manufactured, extracts with high phenol contents increase the detection of changes in pH and antioxidants [157], demonstrating that bioactive ingredients such as carotenoids, anthocyanins, and chlorophylls possess strong antioxidant and antimicrobial activity. However, their application is complex since they present high instability under certain environmental conditions, being able to suffer oxidation or degradation by light [158].

Bakhshizadeh et al. developed an intelligent film based on chitosan nano-fibre (CNF) and β-CD: corn poppy (CP) for monitoring shrimp deterioration. The addition of smart packaging significantly reduced the water solubility from 96% to 42%. The results showed that, during storage, the film changed from coral to gold due to changes in pH (8.3 to 10.5) and the release of ammonium vapors due to protein decomposition. Demonstrating that this intelligent film could effectively be applied to marine products to monitor their shelf life [138].

#### 5.2.4. Spoilage Indicators

Microbial deterioration and the reactions of the food impact freshness because of metabolites that degrade the compounds present, producing off-flavors and sensory rejection. Wei et al. developed a colorimetric sensor to detect the bacterium Salmonella typhimurium (S. Typhimurium) from hexadecyl trimethyl ammonium bromide (CTAB) and an intelligent packaging of β-CD: capped gold nanoparticles (β-CD-AuNPs). This results in supramolecular aggregation accompanied by a color change. In milk samples, the recovery was higher than 93%, which suggests its vital application in the food field [145]. Another optical sensor type is biosensors, which send signals through a receiver to be translated and give an electrical response. Sun et al. design intelligent packaging based on 6G-adamantanamine and β-CD on a nonwoven polyethylene terephthalate (NPET) support, used as a sensor to measure food quality through an irreversible fluorescence change. β-CD, in addition to acting as the intelligent packaging for 6G-adamantanamine, also enhanced the fluorescence response.

#### 5.2.5. Electrochemical Sensors

Using sensors in food is essential to avoid adverse effects on consumers’ health. Electrochemical sensors determine the electro-activity of the analytes present in food that may be the cause of contamination. They are based on redox reactions on electrode surfaces, resulting in electrical signals [127,159].

Zhao et al. developed an intelligent packaging with β-CD and ginkgo nut-derived porous carbon (GNDPC) to incorporate it into a glassy carbon electrode (GCE)-based lattice for the recovery of the pesticide methyl parathion (MP), designing an electrochemical sensor. β-CD increased the dispersibility of GNDPC and improved the recognition and accumulation capacity of the MP. The synergy of this intelligent packaging showed a good absorption of this pesticide in apple and pear juices, with a recovery of more than 95% [142]. Also, Ahmadi et al. designed an electrochemical sensor to identify food dyes in juices. β-CD and arginine were used with AuNPs on a gold electrode surface. The manufactured sensor showed selectivity to analyze the dyes in the presence of other agents that interfere with the signals. The mass transport mechanisms were diffusion and reaction, which were quasi-reversible. The data obtained from the different juices ensured the potential that the application of this methodology represents for the verification of modified drinks [147].

Electrochemical sensors have been incorporated to detect molecules because of their low manufacturing costs. For this reason, the incorporation of CDs into these types of sensors has sought to combine them with novel materials that present good synergy between them. Yun et al. carried out an intelligent packaging applied to capsaicin based on GCE modified with β-CD and reduced graphene oxide (rGO). β-CD was found to have a higher degree of charge transfer. The packaging based on β-CD/rGO/GCE recovered over 94% in the quantification of red pepper oil [146].

GCE has been used for different applications, including adsorbents for dye separation. In addition, they present excellent electrical and mechanical properties, which provide stability to the compounds that are applied to them [160]. Chen et al. designed a conductive molecularly imprinted gel (CMIG) using cationic guar gum (CGG), chitosan β-CD, and multiwalled carbon nanotubes (MWCNTs) by magnetic stirring in a single vessel at low temperature. β-CD enhanced the adsorption of CMIG. Subsequently, the CMIG was brought into contact with a GCE surface. AM extraction was carried out on powdered milk and white vinegar samples, and recoveries greater than 88% were obtained. This research demonstrated the correct application of an electrochemical sensor, which could detect other agents [143].

The use of MWCNTs, compared to other nanocarriers, is more highly effective for releasing compounds due to their physical-chemical properties [161]. An example is the study by Gu et al., where they created an electrochemical sensor with β-CD and MWCNTs to determine the content of capsaicinoids in soy sauce and meat. The results showed that β-CD played a significant role in causing the dispersal of MWCNTs on the GCE surface. The recovery rates were higher than 83%, showing the correct application for detecting trace remains [93]. Similarly, Avan and Filik designed intelligent packaging to detect vitamins (A, D3, E, and K) in aqueous media of micellar solutions based on MWCNTs, β-CD, and GCE, where it was found that β-CD, due to the interaction with MWCNTs, presented high selectivity for soluble vitamins [144].

## 6. Conclusions and Future Trends

The widespread use of CD as packaging in food systems has generated many technological advances in the industry in recent years. Improving the organoleptic properties of food is an indisputable advantage provided by the well-defined structure of CD. Its stoichiometric arrangement allows it to combine with different molecules, enhancing its characteristics without altering its function.

The potential application lies in its ability to protect the guest molecules housed in its cavity, increasing its shelf life by stabilizing packaging and protecting the compounds from external factors such as decomposition due to temperature, oxidation, and photosensitivity. Additionally, this container based on the CD polymer acts as a transport medium, increasing the solubility of the encapsulated compounds and allowing them to be stably used as dry powders. Among the other qualities present in CD, they are most responsible for improving the sensory quality of food by hiding unwanted odors and flavors. In addition, they reduce the effects of evaporation, delay the action of compounds that change food coloration, and thus deliver friendlier flavors to consumers.

Furthermore, it can still have adverse effects related to kidney failure due to its irritant effect. However, its toxicity remains low, and it is accepted by international organizations in charge of human health and governs the use of additives. Therefore, it is applied in low concentrations in the food and pharmaceutical fields since the qualities that offer this type of packaging are higher. As a result, healthier and more functional products are obtained, an option to consider when you want to improve properties in the food, pharmaceutical, chemical, cosmetic, and textile industries, among others. CD’s opportunities in the food industry are remarkable and copious, as consumers demand ever higher standards and companies compete to improve their products to deliver higher quality.

## Figures and Tables

**Table 2:** Most frequently used techniques for structural characterization.

CDs	Guest Molecule	Technique	Results	References
β-	Curcumin	SEM, FT-IR, TGA, UV-vis spectra	SEM: pores disappeared attributed to CD. FT-IR: no new chemical bonds were broken or formed. TGA: did not present water loss.	[62]
β-	Sage essential oil	FT-IR	FT-IR: peak attributed to SEO disappeared.	[75]
β-	Eucalyptus essential oil	FT-IR, DSC, TGA, SEM	FT-IR: blue-shifted. DSC and TGA: improve the stability of EEO and retard the volatilization. SEM: The structure of the particle has transformed, resulting in a smooth surface of the crystal structure	[76]
β-	p-Anisaldehyde	SEM, XRD, TGA, FT-IR	SEM: size reduction and rhomboid crystals in shape. TGA: protection of the inclusion structure for the volatile compounds. FT-IR: peaks disappeared. XRD: new sharp peaks.	[77]
β- HP-β-	(-) borneol	DSC & TG, FT-IR, SEM, XRD, NMR	Comparative study. Promising results demonstrated by TG analysis.	[78]
β-	Rosemary essential oil	FT-IR, TGA-DSC	FT-IR: signal of the oil constituents appeared in the capsule spectrum. TGA-DSC: yeast reduction	[79]
β-	d-limonene	SEM, FT-IR (ATR), TGA, DSC	SEM: particles appeared homogeneously distributed. DSC: prevents the loss of the volatile essential oil.	[80]
β-	Ginger essential oil	SEM, DSC, TGA, FT-IR, XRD	FT-IR: redshift. XRD: disappearance and formation of diffraction peaks and the intensities changed. TGA: thermal protection for GEO.	[81]
β-	Peppermint oil	FT-IR	Confirmation of the formed structure.	[82]
β-	Concentrated orange oil	SEM, FT-IR	SEM: differences in shape and size. FT-IR: changes in spectra.	[83]
α	Benzyl isothiocyanate (BITC)	FT-IR, XRD	FT-IR: slight wavelength shifts XRD: formation of amorphous complex.	[84]
γ-	BITC, phenethyl isothiocyanate (PEITC), and 3-methylthiopropyl isothiocyanate (MTPITC)	FT-IR, TGA, XRD	TGA: elevated temperature required for the complete decomposition of ITCs. XRD: Sharp peaks appeared.	[73]
γ-	Watermelon flavor	SEM, FT-IR, DSC, XRD	SEM: particles smaller than CD. FT-IR: peaks of watermelon disappeared. DSC: higher temperature for evaporation.	[74]
γ-	thymol	SEM, XRD, FT-IR, NMR, DSC, TGA	TGA: improved by hydrogen bonding. XRD: characteristic diffraction peaks disappeared. SEM: smooth surface.	[85]

**Table 3:** Studies of cyclodextrins (CDs) as active packaging (AP).

Packaging	Enhanced Properties	References
PLA/β-CD:AITC	Increase storage of High-Fat Fruit and Vegetable Increases solubility Increases absorption Increases release rate	[108]
LLDPE/β-CD:D-limonene	Prevents the loss of the volatile compounds Antibacterial and antifungal activities Protects from oxidation	[80]
PLA/β-CD-thymol	Prolongs shelf-life in one week for blackberries and raspberries Microbial inhibition Decreases in weight loss Reduces changes in color	[116]
CNC/zein:catechin:β-CD	Inhibits oxidation of soybean oil Prolongs shelf-life due to mechanical properties	[69]
Poly(ethylene oxide)/Tea tree oil:β-cyclodextrin	Microbial inhibition against *E. coli* O157:H7, tested on beef	[117]
PET/Palmarosa EO/β-cyclodextrin	Extends apple shelf life Inhibits the fungus *P. expansum*	[118]
Cellulose nanocrystals/Curcumin, Carvacrol:β-cyclodextrin	Microbial inhibition against *B. subtilis*.	[119]
Cardboard box/Carvacrol, oregano, and cinnamon Eos:β-cyclodextrin	Increase the shelf life of mandarins, inhibiting psychrophiles, mesophiles, enterobacteria, molds, and yeast.	[120]
Chitosan/β-cyclodextrin citrate/oxidized nanocellulose/Clove EO:β-cyclodextrin	Microbial inhibition against *E. coli* and *P. aeruginosa*	[121]
Gelatin/Galangal root oil:β-cyclodextrin	Microbial inhibition against *E. coli* O157:H7 in beef.	[122]
PHBV/Oregano EO:(α-CD and γ-CD)	Antimicrobial activity against *S. aureus* and *E. coli*.	[123]
PVA/Cinnamon EO/β-cyclodextrin: β-cyclodextrin	Microbial inhibition against *E. coli* and *S. aureus* in mushrooms.	[124]

PLA: poly lactic acid; AITC: allyl isothiocyanate inclusion complexes; LLDPE: linear low-density polyethylene; CA: cinnamaldehyde; CNC: cellulose nanocrystals, PET: polyethylene terephthalate, EO: essential oil, PHBV: poly(3-hydroxybutyrate-co-3-hydroxyvalerate); PVA: polyvinyl alcohol.

**Table 4:** Studies of cyclodextrins (CDs) as intelligent packaging (AP).

Packaging	Sensor	Indicator	References
Chitosan/montmorillonite:β-CD	Oxygen	Colorimetric (changing the color from colorless to blue)	[137]
CNF/CP:β-CD	pH	Colorimetric (changing the color from coral to gold)	[138]
PVA/β-CD/acylated roselle anthocyanin	Freshness	Colorimetric	[139]
Chitosan/PVA/curcumin:β-CD	Freshness	Colorimetric	[140]
Rhodamine 6G-adamantamine and β-CD	Shelf-life	Fluorescence	[141]
GCE/GNDPC:β-CD	Electrochemical	Recovery (MP)	[142]
Chitosan/cation guar gum/MWCNTs/β-CD/GCE	Electrochemical	Recovery (amaranth)	[143]
MWCNTs/β-CD/GCE	Electrochemical	Recovery (capsaicin)	[93]
MWCNTs/β-CD/GCE	Electrochemical	Quantification (vitamins)	[144]
β-CD: AuNPs	Spoilage	Colorimetric	[145]
rGO/GCE/β-CD	Electrochemical	Quantification (capsaicin)	[146]
AuNPs:β-CD/arginine	Electrochemical	Quantification (colorant)	[147]

CP: corn poppy; CNF: chitosan nanofiber; PVA: polyvinyl alcohol; MP: methyl parathion; GCE: glassy carbon electrode; GNDPC: ginkgo nut-derived porous carbon; MWCNTs: multi-walled carbon nanotubes; AuNPs: capped gold nanoparticles.
